# Bioremoval and Detoxification of the Anticancer Drug Mitoxantrone Using Immobilized Crude Versatile Peroxidase (icVP/Ba) *Bjerkandera adusta* CCBAS 930

**DOI:** 10.3390/biology11111553

**Published:** 2022-10-23

**Authors:** Kamila Rybczyńska-Tkaczyk

**Affiliations:** Department of Environmental Microbiology, Faculty of Agrobioengineering, The University of Life Sciences, Leszczyńskiego Street 7, 20-069 Lublin, Poland; kamila.rybczynska-tkaczyk@up.lublin.pl

**Keywords:** cytostatic drug, versatile peroxidases, immobilization, genotoxicity, biotoxicity, phytotoxicity

## Abstract

**Simple Summary:**

The study presents relevant research in the fields of environmental microbiology, waste manage-ment and toxicology. Industrial pollutants released into the environment, and climate change are causing significant deterioration of water quality. Oxidoreductases, synthesized during secondary metabolism by white-rot fungi have been applied in many industries, and are particularly frequently used for soil and water environment bioremediation and xenobiotic biodegradation. Immobilization methods are applied to improve the stability and recyclability of the biocatalyst compared to the free enzyme. Immobilization, mainly enzyme immobilization, reduces the number of processing steps due to the easy separation of the biocatalyst itself from its reaction mixture, retention of catalytic activity, and the resulting high degree of reusability and reduction of application costs. Therefore, the aim of this study was to analyze the possibility of bioremoval of the anticancer drug mitoxan-trone (MTX) by immobilized crude versatile peroxidase (cVP/Ba) synthesized by *B. adusta* CCBAS 930. Since biological methods should be characterized not only by efficient biodegradation of xe-nobiotics, but also by a reduction of their toxicity, phyto-, bio-, and genotoxicity, assays were applied to assess the detoxification of mitoxantrone by immobilized cVP/Ba.

**Abstract:**

The aim of this study was to evaluate the biodecolorization and detoxification of the anticancer drug mitoxantron (MTX) by immobilized crude versatile peroxidase of *Bjerkandera adusta* CCBAS 930 (icVP/Ba). The concentrated crude VP was obtained from *B. adusta* CCBAS 930 culture on medium with MTX (µg/mL) addition, immobilized with 4% sodium alginate. MTX removal degree (decolorization), levels of phenolic compounds and free radicals were determined during MTX biotransformation. Moreover, the phytotoxicity (*Lepidium sativum* L.), biotoxicity (multi-species microbial assay, MARA), and genotoxicity (SOS Chromotest) of MTX were evaluated before and after the biological treatment. The use of icVP/Ba (95 U/mL) significantly shortened the bioremoval of 10 µg/mL MTX (95.57% after 72 h). MTX removal by icVP/Ba was correlated with an 85% and 90% decrease in the levels of phenolic compounds and free radicals, respectively. In addition, the use of icVP/Ba contributed to a decrease in the phyto-, bio-, and genotoxicity of MTX. This is the first study to describe the possibility of removing MTX using immobilized crude fungal peroxidase.

## 1. Introduction

Cytostatic drugs are a heterogeneous group of pharmaceuticals used to treat cancer and other diseases through inhibition of cell division, and thus tumor development. One of the main groups of cytostatics are anthracyclic antibiotics (daunomycin, doxorubicin, mitoxantrone), which have been used in cancer therapy for almost 50 years [1]. Mitoxantrone (MTX) is a synthetic antineoplastic drug, structurally analogous to such anthracyclines as doxorubicin. MTX has been used in the treatment of various cancers, including leukemia, non-Hodgkin lymphoma, and breast cancer [2]. Anticancer drug residues have been found in hospitals, domestic and industrial wastewaters, as well as surface waters [3]. Like other pharmaceuticals, both initial MTX and its metabolites are excreted and discharged to the sewage system via urban or hospital effluents. MTX is excreted in 6–11% by urine and in 25% by feces, and the rest is removed in a metabolized form. There are no studies that report the stability of mitoxantrone in water, but according to known findings, the half-life of MTX is up to 9 days [4]. Even low concentrations of pharmaceuticals can be potentially risky for the environment, as they are designed to cause biological effects at low doses [5]. Moreover, pharmaceuticals are likely bioaccumulated by organisms; due to the possibility of increasing their concentration in subsequent links of the trophic chain through biomagnification, even small concentrations of pharmaceuticals in surface waters pose a threat to human and animal health [6]. They are resistant to conventional wastewater treatment technologies and create a potential risk to the environment and human health. Many cytostatic drugs are cytotoxic, teratogenic, genotoxic, and carcinogenic even at low concentrations [7,8,9]. Currently, mitoxantrone is removed from the environment using electrochemical methods [10], adsorption and photocatalytic degradation [11], or green synthesis [12]. Enzymatic treatment is a particularly attractive technology for removing pharmaceuticals resistant to conventional treatments [13]. There is a lack of information in the literature regarding the biological treatment of mitoxantrone. To the best of my knowledge, this is the first study that describes the possibility of removing mitoxantrone using a biological method, i.e., immobilized peroxidase. With the development of molecular biology and biotechnology tools, and thus a better understanding of the structure and catalytic properties of enzymes produced by microorganisms, the possibilities of using environmentally friendly biological methods for removing xenobiotics are increasing [14,15].

Oxidoreductases, including peroxidases, synthesized during secondary metabolism by white-rot fungi, have been used in many industries, including pharmacy, medicine, food processing, and cosmetology [16]. There is growing interest in the application of crude and purified extracellular ligninolytic enzymes for pharmaceutical removal [13,17,18]. They are particularly frequently applied in bioremediation of the soil and water environment and xenobiotic biodegradation. Due to their low substrate specificity, fungal peroxidases can biodegrade various aromatic compounds, such as anthraquinone dyes, pesticides, pharmaceuticals, melanoidins or endocrine disruptor compounds [9,17,18,19,20,21,22]. In addition to the well-known Mn-dependent peroxidase (MnP) and lignin-degrading peroxidases (LiP), versatile peroxidase (VP) seems to be a very promising oxidoreductase for use in xenobiotic biodegradation, as it combines the substrate specificity of both fungal peroxidases, i.e., MnP and LiP [23]. Our previous studies characterized a versatile peroxidase decolorizing anthraquinone derivatives produced by the *B. adusta* strain CCBAS 930 (VP/Ba) [24,25]. Recent studies have shown that purification is necessary to characterize and fully understand the mechanism of action of an enzyme; however, crude or partially purified enzymes are used for practical applications [26,27,28]. This is mainly due to the time required to obtain a highly effective enzyme and the possible cost reduction of the application of the proposed method [29]. Crude or partially purified ligninolytic enzymes can be used as low-cost alternatives in environmental applications [30]. Moreover, crude enzymes are often more stable than purified enzymes due to the presence of a mixture of components that confer protection against excessive oxidation and denaturation [31]. Immobilization methods are applied to improve the stability and recyclability of cells or biocatalysts compared to free cells or enzymes. Immobilization by entrapping the cells or enzymes with a permeable polymer network allows the supply of substrates and removal of reaction products [19,32,33]. Enzymes immobilized via entrapment exhibit improved stability due to the intensified control of their microenvironment, and are active in a wide range of temperatures and pH. Enzyme immobilization reduces the number of processing steps due to the facile separation of the biocatalyst itself from its reaction mixture, the retention of catalytic activity, and the resulting significant reusability and reduction of application costs [16]. Moreover, immobilization of the enzyme using a biodegradable polymer makes the process environmentally safe [34]. Enzyme immobilization allows for the lower consumption of energy, water and reagents. Moreover, the possibility to use the immobilized enzyme in several cycles with the same efficiency, as well as longer shelf-life of immobilized enzymes significantly increase the efficiency of the process. From an economic point of view, the use of immobilized enzyme is cost-effective under continuous conditions. On the other hand, operational stability of the enzymes is one of the main problems in continuous processes [35]. Recent studies underlined the role of immobilized peroxidases–H_2_O_2_-mediated systems as versatile biocatalysts for the biodegradation and biotransformation of a wide spectrum of hazardous environmental pollutants [36].

Therefore, the aim of the present study was to evaluate the effective bioremoval of the anticancer drug MTX by immobilized crude versatile peroxidase (icVP/Ba) synthesized by *B. adusta* CCBAS 930. Since biological methods should be characterized not only by efficient biodegradation of xenobiotics, but also the reduction of their toxicity, the content of phenolic compounds and free radicals was determined along with phyto-, bio-, and genotoxicity assays to assess MTX detoxification with icVP/Ba.

## 2. Materials and Methods

### 2.1. Chemicals

Mitoxantrone hydrochlorine (≥90%), 2,6-dimethoxyphenol (99%) (2,6-DMP), nitrotetrazolium blue (99%) (NBT), protocatechuic acid (97%), sodium alginate, calcium chloride (93%), 30% hydrogen peroxide and malonic acid (99%) were purchased from Sigma-Aldrich (St. Louis, MO, USA). All other chemicals and reagents were of analytical grade.

### 2.2. Bjerkandera Adusta Strain CCBAS 930 and VP Peroxidase Production Profile

The anamorphic *B. adusta* strain CCBAS 930 was isolated as *B. adusta* R59 from black earth soil (Pheozems, FAO). The identification sequences of the rRNA gene fragments: ITS1, 5,8S rRNA and ITS2 of *B. adusta* CCBAS 930 are available in GenBank (AY 319191). The culture was deposited in the Culture Collection of Basidiomycetes Prague, Czech Republic as *B. adusta* CCBAS 930 [37]. The experiments were conducted under stationary conditions (20 days, 26 °C) in 100 mL of liquid mineral medium [24] supplemented with 0.25% glucose and MTX (10 µg/mL). The inoculum was 2 mL (5.0 × 10^7^ cfu) of homogenized 10-day idiophasic mycelium of *B. adusta* CCBAS 930 grown on glucose-potato medium (PDA) [24]. VP peroxidase activity were determined periodically (day 3, 7, 10, 14, 18 and 20) with 20 mM 2,6-dimethoxyphenol (2,6-DMP) in the presence/absence of Mn^2+^ and 0.2 mM H_2_O_2_ at different pH (3.0 and 4.5) [24].

### 2.3. MTX Biodecolorization by Crude Immobilized VP Peroxidase Produced by B. adusta CCBAS 930 (icVP/Ba)

#### 2.3.1. Induction and Partial Purification of VP Peroxidase by *B. adusta* CCBAS 930

In order to obtain VP, the mycelium of the cultured fungus *B. adusta* CCBAS 930 (maximum VP production) was separated on day 14 from the culture fluid by filtration through sterile 0.22-µm syringe filters. In the next step (200 mL of culture fluid), protein precipitation from the solution was performed by salting-out to 80% saturation with ammonium sulfate (NH_4_)_2_SO_4_. The solution was then centrifuged at 7000 rpm/min for 20 min at 4 °C to separate the precipitated protein. The protein pellets were dissolved in 5 mL of 50 mM malonate buffer pH-4.5 and concentrated on the membrane (Amicon Ultra-15 System, Millipore 30 kDa) by centrifugation at 4000 rpm/min for 20 min at 4 °C. In order to evaluate the efficiency of the concentration process, protein content [38] and VP activity were determined in the presence of 20 mM 2,6-DMP [24]. Process efficiency was assessed by comparing the protein content and VP activity before and after concentration. Concentrated crude VP peroxidase was used for immobilization.

#### 2.3.2. Immobilization of VP Peroxidase

Concentrated crude VP (cVP) was immobilized using 4% sodium alginate [19]. Briefly, in sterile conditions, 1 mL of cVP/Ba with an activity of 95 U/mL was added to 9 mL of 4% sodium alginate and mixed (30 °C, 130 rpm/min). After 20 min, alginate with VP was transferred into 50 mL of 0.2 M CaCl_2_ using a sterile syringe. Subsequently, the immobilized crude VP peroxidase (icVP/Ba) was incubated at 4 °C for 2 h.

#### 2.3.3. MTX Bioremoval by icVP/Ba

Immobilized crude VP peroxidase was mixed with 50 mL of MTX solution (10 µg/mL) in 50 mM Na-malonate buffer (pH = 4.5) with 0.2 mM H_2_O_2_ and 0.1 mM MnSO_4_ in 50 mM Na-malonate buffer (pH = 4.5) and agitated at 130 rpm/min and 30 °C for 72 h. Decolorization was assessed by periodic measurements of absorbance (A630 nm) of the supernatants [39]. Moreover, measurements in the visible spectrum range from 300 to 700 nm were performed during MTX treatment with icVP/Ba.

### 2.4. Phenolic Compound (PhC) and Free Radical (SOR) Contents

PhC content was assessed at A740 nm according to Singleton and Rossi (1965) [40], with a slight modification [41]. SOR content was estimated at A560 nm with nitrotetrazolium blue (NBT) as a substrate [42]. As control, 50 mM Na-malonate buffer (pH = 4.5) with MTX at a concentration of 10 µg/mL was used.

### 2.5. Evaluation of Phyto-, Bio- and Genotoxicity

Phyto-, bio- and genotoxicity assays were applied to determine acute toxicity and its long-term effects on cultures untreated and treated with MTX. The Phytotestkit (Tigret, Poland) was used to determine the direct effects on germination and growth of young *Lepidium sativum* L. roots in comparison to controls (distilled water) in the reference soil. The phytotoxicity assay was performed according to the producer’s protocol with assessment of root growth inhibition (RGI) and germination index (GI) [43]. The data on RGI and GI of MTX before treatment were derived from the study by Rybczyńska-Tkaczyk (2021) [39].

The Multi-species microbial assay (MARA), based on the activity of 11 living microorganisms and their ability to reduce colorless 2,3,5-triphenyltetrazoline hydrochloride to red formazan, was performed to determine biotoxicity after MTX biotransformation by icVP/Ba [39]. The data on biotoxicity of the MTX before treatment were derived from the study of Rybczyńska-Tkaczyk (2021) [39]. The results were processed using an image analysis program to facilitate the calculation of the MTC (microbial toxic concentration) value (% vol.) for each strain.

Genotoxicity analysis was performed using the SOS ChromoTest (distribution Tigret, Poland) using genetically engineered bacteria, *Escherichia coli* PQ37, to detect DNA-damaging agents. In this assay, β galactosidase (β-gal) activity is indicative of the degree of SOS induction and bacterial genotoxicity, i.e., the induction factor (IF). In addition, bacterial alkaline phosphatase (AP) activity was used to determine bacterial cytotoxicity, i.e., the reduction factor (RF). The ratio of β-gal/AP activity was defined as the corrected induction factor (CIF = IF/RF), and it was used to indicate the degree of SOS induction for the tested samples [39]. Briefly, overnight bacterial cultures were grown in fresh LB medium (20 g tryptone/L, 10 g yeast extract/L, 20 g sodium chloride/L, pH 7.4) to an optical density of 600 nm = 0.5 and subsequently mixed (*v*/*v*) with the tested samples and incubated for 1.5 h at 37 °C); distilled water was used as a negative control. Positive control included 4-nitroquinoline-1-oxide (4NQO) as a mutagen and 2-aminoanthracene (2AA) as a pro-mutagen with the S9 fraction (metabolic activation). The β-gal (A620 nm) and AP (A405 nm) activities were determined in a 96-well plate reader (EPOCH, Biokom). Significant genotoxic activity was defined as a corrected induction factor (CIF) equal to or greater than 1.2 [39].

### 2.6. Statistical Analysis

Data are presented as the means ± standard deviation (SD) of three independent experiments. Data were analyzed using one-way analysis of variance (ANOVA) followed by Tukey’s multiple comparison assay. Statistical analysis of microbial growth and percentage growth inhibition of microorganisms were carried out using the MARA software in each well relative to the respective control as well as MTC values.

## 3. Results

### 3.1. VP Production and Partial Purification

**Figure 1:**
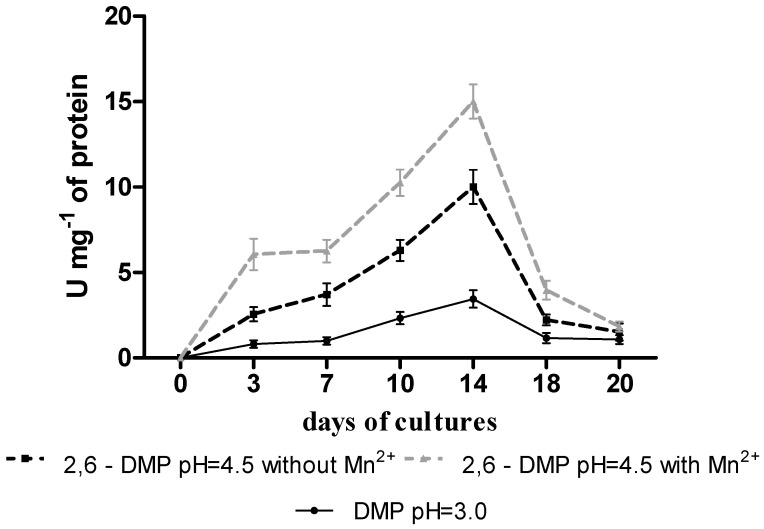
Production profile of versatile peroxidase (VP) in stationary cultures of *B. adusta* CCBAS 930 with 10 µg/mL of mitoxantrone (MTX) in the presence of 2,6-dimethoxyphenol (2,6-DMP) as a substrate.

The VP peroxidase production profile in the presence of MTX (10 µL/mL) showed the highest activity (3.45–15 U/mg) on day 14 of cultivation of the fungus *B. adusta* CCBAS 930 (Figure 1). On day 14 of cultivation, the post-culture fluid was separated from the mycelium and used to purify VP. The highest protein content was recorded after ultrafiltration of the post-culture fluid (38.57 µg/mL). The protein content in the concentrated post-culture fluid increased by 52% compared to the initial variant (18.66 µg/mL). The activity of VP peroxidase was found both in the original and partially purified supernatants. Before culture fluid concentration, the highest VP activity was found in the buffer with pH = 4.5 and Mn^2+^ ions (137.72 U/mg). After partial purification, the highest VP peroxidase activity was recorded in the buffer with pH = 4.5 and Mn^2+^ ions (2502.10 U/mg). The concentrated post-culture fluid was characterized by over 18-fold higher specific VP activity compared to the initial post-culture fluid (Table A1).

### 3.2. Bioremoval of Mitoxantrone by icVP/Ba

**Figure 2:**
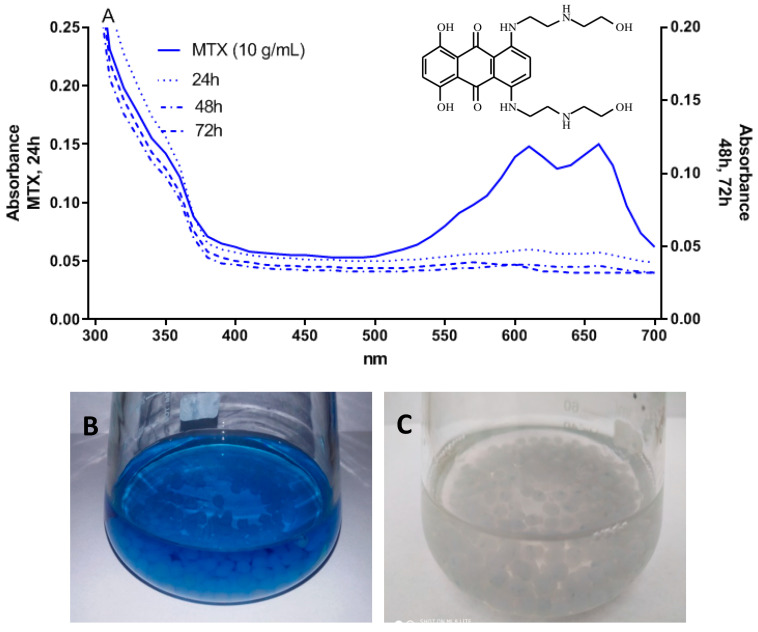
UV-visible spectra (**A**) of initial MTX solution (10 µL/mL) before (**B**) and after 72-h treatment by icVP/Ba produced by *B. adusta* CCBAS 930 (**C**).

To evaluate the decolorization efficiency of icVP/Ba, the degree of MTX solution decolorization was measured after 24, 48 and 72 h. A significant rate of MTX removal was observed in the experimental setup with icVP/Ba. Efficient MTX decolorization was noted after 24 h (23%), and after 48 h, a significant increase in the degree of decolorization was observed (75%). The highest degree of MTX removal (95.57%) was recorded after 72 h (Figure 2A). We did not observe any colorization of Ca-alginate beads (blue) upon contact with MTX (Figure 2B,C).

### 3.3. PhC and SOR Contents

**Figure 3:**
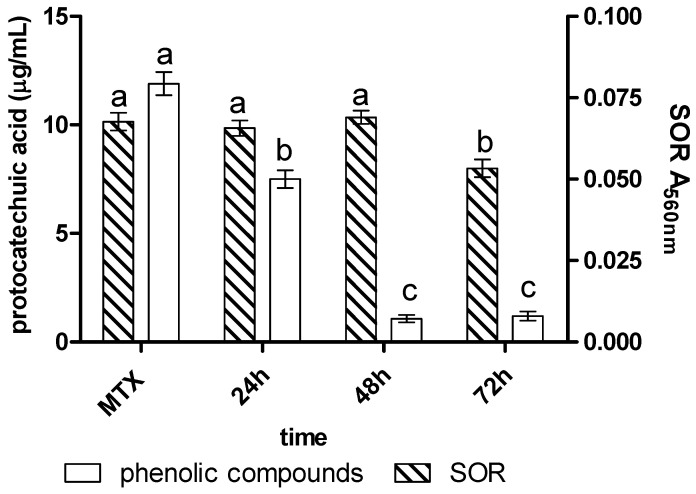
Content of phenols (PhC) (µg/mL protocatechuic acid) and free radical levels (SOR) (A560 nm) during MTX (10 µg/mL) treatment with icVP/Ba; different lowercase letters a–c indicate significant differences at α = 0.05.

During the biotransformation of MTX by icVP/Ba, a gradual decrease in the content of PhC and SOR was recorded. After 24 h, an 85% decrease in the content of PhC was observed, and it remained constant until the end of the experiment. The lowest content of these compounds was recorded after 72 h of treatment (1.04 µg/mL). With respect to SOR, we observed a decrease in its concentration during MTX biotransformation by icVP/Ba with the highest reduction after 48–72 h (more than 90%) (Figure 3).

### 3.4. Detection of Phyto-, Bio-, and Genotoxicity before and after MTX Treatment with icVP/Ba

**Figure 4:**
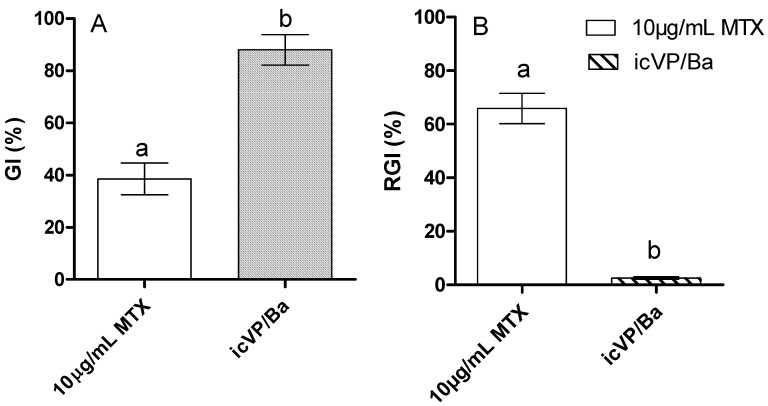
Phytotoxicity of initial MTX solution (10 µg/mL) [39] and after 72-h treatment with icVP/Ba; GI–germination index (**A**), RGI–root growth inhibition (**B**); different small letters are significantly different at α = 0.05 compared to control (MTX 10 µg/mL).

**Figure 5:**
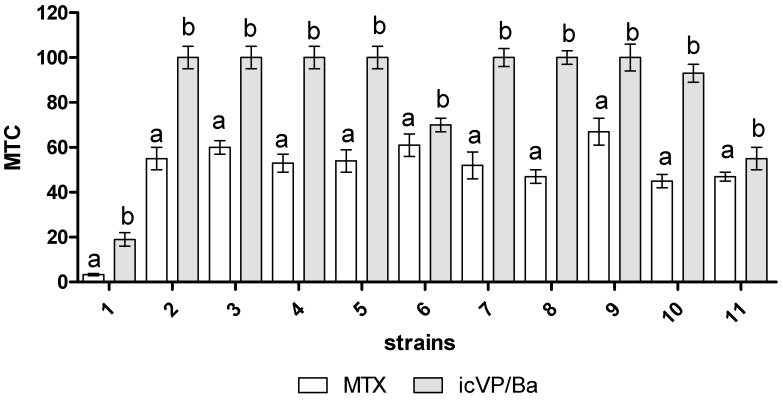
Microbial toxic concentration value (MTC %) for each strain (1) *Microbacterium* sp., (2) *Brevundimonas diminuta*, (3) *Citrobacter freudii*, (4) *Comamonas testosteroni*, (5) *Enterococcus casseliflavus*, (6) *Delftia acidovorans*, (7) *Kurthia gibsoni*, (8) *Staphylococcus warneri*, (9) *Pseudomonas aurantiaca*, (10) *Serriatia rudidaea*, and (11) *Pichia anomala* before MTX (10 µg/mL) [39] and after 72-h treatment with icVP/Ba; different lowercase letters indicate significant differences at α = 0.05.

Significant differences (*p* < 0.05) were found in the inhibition of *L. sativum* seed germination after MTX treatments with icVP/Ba, i.e., GI = 86.50 ± 7.20 (Figure 4A). With regards to root growth inhibition (RGI), a significantly increased root growth of *L. sativum* was observed after the application of cVP/Ba-treated MTX (Figure 4B). The degree of MTX biotoxicity and genotoxicity after treatment with icVP/Ba was estimated using the multi-species MARA assay and SOS ChromoTest based on modified *Escherichia coli* PQ37. A wide range of MARA species sensitivity was recorded before and after MTX treatment with icVP/Ba, with the MTC min. values for the most sensitive species, *Microbacterium* sp., amounting to 3.3% and 19.0%, respectively. After treating 10 µg/mL MTX solution with icVP/Ba, the average MTC values increased from 49.48% to 85.18% (only low selective toxicity) (Figure 5). MTX genotoxicity before and after icVP/Ba treatment was dose-dependent and decreased with increasing sample dilutions. The highest MTX genotoxicity before treatment was found for 2.5 and 1.25 µg/mL concentrations, with the CIF factor of 3.05 and 2.20, respectively (Table A2). Thus, the results indicated that MTX before treatment with icVP/Ba was genotoxic at lower concentrations (2.5 and 1.25 µg/mL), and cytotoxic at higher concentrations (5 and 10 µg/mL). After icVP/Ba treatment, the CIF factor was <1.2, and the genotoxicity of the samples was on average 30% lower. We did not observe any genotoxicity (pro-mutagenic activity) with metabolic activation (with the S9 fraction; data not shown).

## 4. Discussion

Many irrigated areas around the world are experiencing water shortages due to climate change, and surface and groundwater pollution. Water scarcity creates serious economic and social concerns. In view of decreasing clean water resources and increasing pollution of the aquatic and soil environment, sustainable solutions are sought for the effective removal of xenobiotics. The reuse of wastewater in agriculture is gaining increasing acceptance. It is an agronomic option that is increasingly explored and applied in regions with water scarcity, growing urban populations, and rising demand for irrigation water. Work is still underway to improve the efficiency of biological methods for recycling municipal and industrial wastewater, which will facilitate its reuse, e.g., for agricultural irrigation [44,45]. In order for industrial or municipal wastewater to be used for agricultural irrigation, it must meet appropriate sanitary requirements and must not contain toxic substances [45,46].

The use of a microorganism or its enzymes for the biodegradation of xenobiotics is less time consuming, produces fewer or no toxic secondary products and is a good economic solution compared to other conventional technologies [13]. However, the use of enzymes for biodegradation has more advantages than the use of microorganisms [34,47,48,49]. Enzymes are less likely to be inhibited by chemicals that may be toxic to living organisms, and their cost could eventually be lower than that of other methods if commercially available enzymes are produced in bulk quantities. Moreover, enzymes work in a wide range of aromatic compounds and require low retention times in relation to other treatment methods [47]. However, peroxidases are inactivated by high levels of H_2_O_2_, free radicals, as well as oligomeric and polymeric products formed during the reaction. Therefore, suitable techniques have been introduced, such as immobilization, to reduce the extent of peroxidase inactivation in the presence of inhibitors, including excessive H_2_O_2_ levels, as well as to improve stability, reusability, and catalytic capacity of peroxidases [34,47,48,49]. Immobilized fungal peroxidases, compared to free enzymes, have a high application potential for removing hazardous contaminants. On the other hand, even well-characterized enzymes still have large untapped potential for biodegradation applications. Current research on immobilized fungal enzymes is restricted to the treatment of well and long known pollutants, such as phenols, industrial dyes, lignin, pharmaceutics or pesticides. In order to fully exploit the possibilities of immobilized peroxidases, they should also be used to remove new hazardous pollutants, such as 2-amino-4-nitrotoluene, 2,4-diaminotoluene and tertiary butyl alcohol [49].

The properties of enzymes produced by fungi allowing to degrade industrial wastewater, especially oxidoreductase enzymes, due to their known oxidizing potential for high redox compounds, e.g., industrial dyes, post-industrial lignin, melanoidins, and pesticides, have been known for a long time [18,19,43,50]. In recent years, an increasing number of studies have described the possibility of using fungal oxidoreductases to remove pharmaceuticals, such as antibiotics (sulfamethoxazole, tetracycline), antidepressants (citalopram hydrobromide and fluoxetine hydrochloride), antiepileptics (carbamazepine), anti-inflammatory drugs (diclofenac and naproxen), estrogen hormones (estrone, 17β-estradiol, 17α-ethinylestradiol), and cytostatic drugs (daunomycine, doxorubicyne, bleomycin, vincristine) [9,51,52,53,54] Ligninolytic enzymes obtained from white-rot fungi are non-specific for organic compounds. They use a free radical mechanism to catalyze the degradation of a wide range of pollutants. A new area of application of these enzymes is the possibility of using them for biodegradation of pharmaceuticals [9,13]. However, the main disadvantage of free enzymes is their limited application in large-scale processes. Moreover, free enzymes are difficult to separate from the medium, which reduces their reusability and increases costs. To overcome such limitations, immobilization of enzymes is one of the most effective methods improving their properties [35], applied in enzymatic biodegradation of pharmaceuticals [16,55]. The use of immobilized enzymes for biodegradation of xenobiotics reduces the time and improves efficiency of their removal compared to conventional microbial cultures. Previous data indicated an effective MTX removal (over 90%) after 21 days of stationary cultures of *B. adusta* CCBAS 930 (data not shown). Other anthracycline antibiotics, i.e., daunomycin and doxorubicin were removed (80–90%) after 3-week cultures of this fungus [9]. In this study, a significant increase in MTX removal (75%) was observed after 48 h, with the highest removal rate (95.57%) after 72 h. As suggested in a previous study, removal of xenobiotics using Ca-alginate-immobilized enzyme may be based on biodegradation or bioaccumulation/biosorption of compounds onto alginate beads [56]. In the present study, we did not observe colorization of alginate beads (blue) after contact with MTX. This observation demonstrated that the mechanism of MTX removal was associated with immobilized cVP/Ba biodegradation. The UV-Vis scan spectrum (200–800 nm) of the supernatants indicated that MTX color removal at 630 nm (maximum absorbance) decreased with time. Moreover, no additional peaks were observed, suggesting efficient biodegradation of MTX by cVP/Ba.

Recently, VP peroxidase has gained interest as an elimination enhancer, and an increasing number of studies have described the possibility of using VP for biodegradation of various xenobiotics, e.g., pharmaceuticals and endocrine disrupting compounds [21,22,51]. Additionally, previous studies indicated that VP immobilization significantly improved biodegradation of pharmaceuticals. Taboada-Puig et al. (2011) [57] and Touahar et al. (2014) [58] demonstrated an increase in the effective removal (>80%) of nonsteroidal anti-inflammatory drugs: acetaminophen, naproxen, mefenamic acid, diclofenac, indomethacin, and endocrine-disrupting chemicals: bisphenol A, nonylphenol, triclosan, 17a-ethinylestradiol, and 17b-estradiol due to cross-linked enzyme aggregates (CLEAs) based on, e.g., VP peroxidase synthesized by *B. adusta* [57,58]. The authors underlined the possibility of using VP for xenobiotic biodegradation, as it has a comparable or even wider spectrum than laccases (Lac) [57,58].

The parameters and catalysts of enzymatic reaction are equally important. The catalytic cycles of peroxidases consist of two-electron oxidation of the native ferric enzyme to compound I by H_2_O_2_ and two single-electron reductions via intermediate compound II to its resting state by appropriate reducing substrates [59]. Due to this function, peroxidases are dependent on H_2_O_2_ as an essential co-substrate in their catalytic cycle. Moreover, H_2_O_2_ may affect the operative stability of peroxidases. Low levels of H_2_O_2_ support stable biocatalytic activity of peroxidases resulting in increased conversion of aromatic compounds, but the required concentration of H_2_O_2_ in a peroxidase-H_2_O_2_-mediated reaction depends on substrate chemical structure and its initial concentration [34]. Therefore, H_2_O_2_ plays a key role in the catalytic cycle of peroxidases, and its addition is very important when peroxidases are directly used in xenobiotic biodegradation [27,28]. Our study indicated that Na-malonate buffer (50 mM, pH = 4.5) in the presence of 0.2 mM H_2_O_2_ created optimal conditions for MTX removal by VP derived from *B. adusta* CCBAS 930. Wen et al. (2010) [28] indicated that during direct tetracycline biodegradation by Mn-dependent peroxidase (MnP) from *Phanerochaete chrysosporium* BKM-F-1767, low H_2_O_2_ levels led to low catalytic activity of MnP. However, if excess H_2_O_2_ is added during tetracycline removal, the structure of MnP may be disrupted. Moreover, VP is characterized by a higher catalytic activity in the presence of low concentrations of Mn^+2^ ions [21,60].

The results also showed that MTX removal by icVP/Ba was associated with a decrease in free radical and phenolic contents. The decrease in the content of phenols during the treatment of anthracyclines by peroxidases indicated biodegradation of these compounds via oxidation. This is an additional advantage of using immobilized peroxidase instead of fungal cultures for the biodegradation of xenobiotics. The treatment of xenobiotics in fungal cultures is accompanied by the production of phenolic compounds and free radicals, as well as biodegradation products, which increase phyto- and biotoxicity of supernatants [9,24,61]. In addition to the efficient removal of xenobiotics, their degradation products must also be safe for the environment. Physical and chemical methods used for the removal of xenobiotics, e.g., pharmaceuticals, are associated with the risk of formation of toxic secondary products, which in some cases are more toxic than the initial compound. Studies on the degradation of anticancer drugs and/or their metabolites (tamoxifen and etoposide) with free chlorine reported up to 110-fold higher potential aquatic toxicity compared to the parent compound, demonstrating an increase in the final effluent toxicity [62]. On the other hand, when using traditional fungal cultures (stationary or agitated) during xenobiotic biodegradation, there is a possibility of biosynthesis of secondary metabolites. They may contribute to masking the efficiency of the biodegradation process and increase the toxicity of post-culture fluids due to the presence of fungal metabolites in addition to the degradation products [61,63].

From a practical point of view, it is possible to introduce pharmaceutical effluents treated with icVP/Ba into the soil without disturbing the ecological balance of soil microorganisms. As previously reported by other authors, treated wastewater can be used for agricultural irrigation, as long as it does not contain toxic substances [44,45]. Therefore, bio-, phyto-, and genotoxicity tests were performed in the present study to evaluate the usefulness of Ca-alginate-immobilized cVP/Ba for MTX biodecolorization. Bacteria and yeasts are suitable organisms for ecotoxicity evaluation due to their small size, rapid growth rate, short generation time and ease of culturing. They also incorporate toxicants in their metabolism faster than higher organisms. Different strains show varied sensitivities to individual chemicals, and this property was utilized in the MARA assay, which combines 11 inhibition tests and provides a toxic fingerprint of the chemicals tested within a single assay [64]. The microbial strains used in the MARA assay belong to typical environmental strains naturally occurring in the soil and water environment [64]. Some of these strains, such as *Pseudomonas* sp., *Microbacterium* sp., and *Comamonas* sp. are involved in the biodegradation of aromatic compounds, e.g., phenols, polychlorinated bis-phenyles and pesticides [65,66,67,68]. Microorganisms can potentially utilize xenobiotic contaminants as carbon or nitrogen sources to sustain their growth and metabolic activities [68]. Therefore, the biotoxic effect of xenobiotics against bacterial strains involved in the biodegradation of these compounds significantly reduces the efficiency of bioremediation of the microflora of the soil and water environment [68]. As described previously, even low concentrations of pharmaceuticals cause biotoxic effects [5]. Pharmaceuticals are classified according to their predicted no-effect concentration (PNEC). PNEC is defined as the pharmaceutical concentration at which no pharmacological effect is expected to occur for a specific organism. Based on the PNEC values, pharmaceuticals are classified into three categories: category 1—compounds with a hazard Quotient (HQ) < 1 or those without eco-toxicological hazards (less ecotoxic at PNEC values > 1 μg/L); category 2—hazardous compounds with HQ values between 1 and 1000 or with intermediate ecotoxicity with PNEC values > 100 ng/L but lower than 1 μg/L; and category 3—highly hazardous compounds with HQs > 1000, including the most ecotoxic compounds with PNEC values < 1 ng/L [5]. Research carried out in this study showed that the acute toxicity (based on MARA assay) was significantly reduced after treating MTX with icVP/Ba. The available ecotoxicological data for MTX are very limited, but previous works indicated that anthracycline antibiotics and their biotransformation products after treatment with immobilized mycelium of *B. adusta* CCBAS 930 showed biotoxic activity only against *Micobacterium* sp. [39].

In addition to the ecological balance of soil microorganisms, the proper growth of plants is also very important. The presence of xenobiotics, e.g., pharmaceuticals in soil can significantly affect the germination and growth of crops and industrial plants. In case of a soil used for agricultural crops, its fertility decreases slowly, which has adverse consequences for agricultural crops both quantitatively, by diminishing production and soil yield, and qualitatively, as a result of the propagation of pollutants from soil to plants, and therefore in the food chain [69]. The current study showed that after MTX biodecolorization with icVP/Ba, its phytotoxicity decreased significantly. Previous studies have reported that during enzymatic biotransformation of aromatic compounds (melanoidins, daunomycin, doxorubicin) using oxidoreductases, especially VP peroxidase, the phytotoxicity of these compounds was significantly decreased. The reduction of phytotoxicity of these aromatic compounds was observed during treatment with immobilized *B. adusta* CCBAS 930 cultures characterized by overproduction of VP peroxidase. Root growth and seed germinations of *L. sativum* L. in the presence of supernatants from 7-day immobilized *B. adusta* CCBAS 930 cultures were significantly increased by 33–40% and 30–85%, respectively [19,39].

In the detoxification of xenobiotics, in addition to acute toxicity, it is also important to determine the potential long-term effects, including genotoxicity. Our results indicated that before biodegradation by immobilized cVP/Ba, MTX was not only genotoxic at lower concentrations (2.5 and 1.25 µg/mL), but also cytotoxic at higher concentrations (5 and 10 µg/mL). Moreover, we recorded reduced MTX genotoxicity after treatment with icVP/Ba. Previous studies showed that other anthracycline antibiotics, including daunomycin and doxorubicin, exhibited strong genotoxic properties as measured by the SOS Chromotest [39]. Therefore, the present results indicated that Ca-alginate immobilization of cVP/Ba not only increased the efficiency of xenobiotic removal, but also their detoxification degree. This is consistent with earlier data indicating a possibility of using immobilized ligninolytic enzymes for pharmaceutical removal and detoxification [70]. *T. versicolor* laccase immobilized on CPC-silica beads efficiently detoxified sulfathiazole and sulfamethoxazole [70].

## 5. Conclusions

Crude VP was immobilized on Ca-alginate beads using the entrapment method to improve its practical efficiency in MTX removal. Considering the application value, partial VP purification makes its potential application more efficient and economical. Reduced phytotoxicity and acute and chronic toxicity assays confirmed MTX biotransformation into non-toxic compounds, clearly proving the usefulness of Ca-alginate-immobilized cVP/Ba for removal and detoxification of MTX. The reduction of phyto-, bio-, and genotoxicity of MTX after treatment with immobilized cVP/Ba is promising from the point of view of its potential application for the removal of cytostatic drugs from wastewater and its subsequent reuse, e.g., for agricultural irrigation. Furthermore, the knowledge gained may lead to the application of icVP/Ba in industrial effluent treatments, especially those containing cytostatic drugs. Further research is required to select the optimal method of cVP/Ba immobilization, which would ensure a higher increase in the efficiency of this process.

## Data Availability

Not applicable.

## Appendix A

**Table A1:** Partial purification efficiency of versatile peroxidase (VP) produced by *B. adusta* CCBAS 930.

Purification Step	Total Volume (mL)	Total Protein (mg)	Total Actvity (U)	Specific Activity (U/mg of Protein)	Purification Fold	Yield (%)
Crude enzyme	200	3.732	514.00 ^a^	137.72	1	100
(NH_4_)_2_SO_4_ precipitation	5	4.18	128.80 ^a^	30.81	0.22	25.06
Ultrafiltration (30 kDa cut off)	1	0.038	95.08 ^a^	2502.10	18.17	18.50

^a^–VP activity in 50 mM Na-malonate buffer pH = 4.5 with Mn^2+^.

**Table A2:** Genotoxicity levels of mitoxantrone (MTX) before and after treatment with immobilized crude peroxidase produced by *B.adusta* CCBAS 930 (icVP/Ba).

Samples	C	V	CIF	SD
MTX before treatment (initial solution 10 µg/mL)	10	-	**1.39**	0.02
	5		0.43	0.04
	2.50		**3.05**	0.06
	1.25		**2.19**	0.04
	0.62		0.17	0.02
	0.31		0.11	0.02
	0.15		0.64	0.05
MTX after treatment with immobilized icVP/Ba	-	100	0.85	0.07
		50	0.98	0.05
		25	0.93	0.05
		12.50	0.76	0.04
		6.25	0.15	0.01
		3.12	0.09	0.02
		1.56	0.10	0.02

C—concentration in µg/mL; V—in %, CIF—corrected induction factor, SD—standard deviation, genotoxic samples are indicated in bold.
